# The Overexpression of TGF-β and CCN2 in Intrauterine Adhesions Involves the NF-κB Signaling Pathway

**DOI:** 10.1371/journal.pone.0146159

**Published:** 2015-12-31

**Authors:** Xiang Xue, Qing Chen, Gang Zhao, Jin-Yan Zhao, Zhao Duan, Peng-Sheng Zheng

**Affiliations:** 1 Department of Gynaecology and Obstetrics, the Second Affiliated Hospital, Xi’an Jiaotong University Medical School, Xi’an, the People’s Republic of China; 2 Department of Reproductive Medicine, the First Affiliated Hospital, Xi’an Jiaotong University Medical School, Xi’an, the People’s Republic of China; Boston University Goldman School of Dental Medicine, UNITED STATES

## Abstract

Intrauterine adhesions (IUA) are a significant cause of menstrual disturbance and infertility, but their pathogenesis still remains unclear. Here, we investigated the expression of TGF-β and CCN2 in IUA endometrial tissue by immunohistochemistry, western blotting and qRT-PCR assays, and found the expression of TGF-β and CCN2 in the endometrial tissue of IUA was significantly increased compared to normal endometrium and uterine septum (P<0.01), suggesting that TGF-β and CCN2 may play an important role in the formation of IUA. Moreover, the activity of the NF-κB signaling pathway in endometrial tissue of IUA was also significantly enhanced compared to normal endometrial and uterine septum (P<0.01) and positively correlated with the expression of TGF-β and CCN2, which suggested that TGF-β and CCN2 expression may be involved in the NF-κB signaling pathway. Blocking the NF-κB signaling pathway using SN50 resulted in the reduced expression of TGF-β in RL95-2 cells, which confirmed the association of the NF-κB signaling pathway and TGF-β in endometrial cells. Additionally, the expression of TGF-β and CCN2 was associated with IUA recurrence, which provides a potential prognostic indictor for IUA. Together, these results demonstrated that TGF-β and CCN2 play an important role in IUA formation, whose mechanism was associated with the activation of the NF-κB signaling pathway.

## Introduction

Intrauterine adhesions (IUA), also known as Asherman syndrome, are a consequence of trauma to the endometrium, producing partial or complete obliteration in the uterine cavity and/or the cervical canal, and are associated with menstrual abnormalities, infertility, recurrent pregnancy loss and other complications later in the pregnancy[1]. Although excessive curettage is considered the primary cause, IUA is known to be associated with diverse non-traumatic factors, such as postabortal sepsis, puerperal sepsis and infections. In recent years, with uterine cavity surgery becoming increasingly common, the incidence of IUA has increased and has become the second most common cause of female infertility[2]. The prevalence of IUA varies by the type of injury and ranges from 16% to 24% in women undergoing pregnancy-related curettage and 31% to 45% after hysteroscopic myomectomy[3], which severely affects women’s health and fertility requirements.

Presently, hysteroscopy is employed for the diagnosis and treatment of IUA and remains the gold standard diagnostic technique because it allows the most accurate confirmation of the presence, extent and nature of IUA[4]. Therefore, although various techniques for adhesiolysis and the prevention of scar reformation have been proposed, hysteroscopic lysis of adhesions is still the main method of treatment. However, an ongoing concern is how to decrease the likelihood of recurrence after surgical repair. It is well established that the formation of IUA likely involves hypoxia, reduced neovascularization, and altered expression of adhesion-associated cytokines, but the exact mechanisms are not well understood. In the process of endometrial repair, the excessive generation of extracellular matrix (ECM) and increasing proliferation of fibroblasts ultimately results in the formation of fibrous scar adhesions. Therefore, the fibroblast and fibrosis play a critical role in the IUA development[5]. Previous studies have reported that the formation of fibrous scar tissue may be associated with the abnormal expression of some cytokines related to tissue fibrosis [6]. TGF-β has long been believed to be a central mediator of the fibrotic response, as this cytokine induces fibroblasts to synthesize and contract ECM[7]. Horbelt et al. reported that TGF-β is associated with liver fibrosis[8]. Connective tissue growth factor (CTGF/CCN2) is a protein found in the extracellular matrix (ECM) and functions as a modifier of adhesive signaling in response to ECM and cytokines[9]. It plays key roles in cell adhesion and migration, as well as in matrix remodeling[10].The overexpression of CCN2 has been observed in wound repair as well as in fibrotic disorders of the skin, kidney, liver and pancreas[11–14]. Previous studies have reported a crosstalk between TGF-β and CCN2, and demonstrated that TGF-β induces CCN2 expression in dermal fibroblasts and mesenchymal cells through Smad3, PKC and the Ras/MEK/ERK pathway[15]. Thus far, however, the role of TGF-β and CCN2 in the IUA formation remains unclear. We hypothesized that TGF-β and CCN2 may be involved in the fibrogenesis of endometrial tissues after injury. Therefore, in this study, we investigated the expression of TGF-β and CCN2 in IUA endometrial tissue.

The NF-κB signaling pathway plays a critical role in many biological processes, including innate immunity, liver inflammation, fibrosis and the prevention of apoptosis[16]. It has been reported that the NF-κB signaling pathway is also involved in fibrotic progression, and TLR4 may promote liver fibrosis through the NF-κB cascade[17]. Notably, the pro-inflammatory cytokine, interleukin-1β (IL-1β) can induce TGF-β expression in lung epithelial cells through the activation of the NF-κB pathway and the promotion of p65 translocation to the nucleus and binding to the TGF-β promoter[18]. These findings demonstrate a correlation between NF-κB signaling pathway and TGF-β expression in fibrogenesis. However, the functional role of NF-κB signaling pathway in IUA is still not fully understood. Here, we further investigated the activity of the NF-κB signaling pathway in IUA endometrium.

It has been reported that the recurrence rate of severe IUA after surgical repair was high (up to 20.0–62.5%)[19, 20], whereas, the rate of IUA recurrence in post-uterine septum resections was less than 1%. Uterine septum is a common type of congenital uterus malformation that is usually associated with recurrent abortion, premature birth, fetal abnormalities and sterility and is one of the most common causes of infertility of women. To explore the mechanism of IUA formation in depth, we also investigated the expression of TGF-β and CCN2 in the endometrium of uterine septum, and further analyzed the relationship between the recurrence rate of IUA and the expression of TGF-β and CCN2.

In this study, TGF-β and CCN2 were found to be overexpressed in IUA endometrial tissue and associated with the activation of NF-κB signaling pathway.

## Materials and Methods

### Patient samples

A total 100 samples were collected from the Second Affiliated Hospital, Medical College of Xi’an Jiaotong University from January 2011 to January 2013. The study was approved by the Ethics Commission of Medical College of Xi’an Jiaotong University and conducted according to the principles of the Helsinki Declaration. Written informed consent was obtained from each participant. 70 endometrial tissues were collected from IUA patients diagnosed by hysteroscopy, and divided into mild, moderate and severe groups according to a modified classification based on the European Society of Hysteroscopy (ESH) and European Society of Gynaecological Endoscopy (ESGE) classification of intrauterine adhesions (Table 1). 15 endometrial tissues were collected from patients with uterine septum, and 15 normal endometrial tissues from patients without IUA and uterine septum who received hysteroscopy due to male infertility or other factors in the same period were used as controls. All of the patients had regular menstrual cycles, did not receive hormone therapy during the three months before surgery, and were not pregnant or lactating during the study. Patients who had additional endometrial complications, including dysfunctional uterine bleeding, adenomyosis, polycystic ovary syndrome, and other hormone-dependent diseases, were excluded. The characteristics of the patients are displayed in Table 2. There were no significant differences in age, weight or parity.

**Table 1:** Classification of 70 patients with intrauterine adhesions.

Classification	Condition	Cases (n)
Mild	Filmy adhesion occupying less than one-quarter of the uterine cavity. Ostial areas and upper fundus minimally involved or clear.	13
Moderate	One-fourth to three-fourths of the cavity involved. Ostial areas and upper fundus partially involved. No agglutination of uterine walls	25
Severe	Greater than three-fourths of the cavity involved. Occlusion of both ostial area and upper fundus. Agglutination of uterine walls	32

**Table 2:** The characteristics of patients with intrauterine adhesions.

Group	n	Age (years)	Weight (kg)	Uterine size	Parity
Normal endometrium	15	30.1±2.3	53.5±4.2	7.4±0.6	2.1±0.5
Uterine septum	15	29.5±1.4	56.12±5.7	6.6±0.9	2.2±0.6
Mild IUA	13	27.1±2.2	54.3±3.4	7.2±1.1	2.2±0.7
Moderate IUA	25	26.8±1.7	55.0±4.0	7.0±1.3	2.4±0.6
Severe IUA	32	28.3±2.1	57.7±5.6	6.7±1.4	2.7±0.4

### Western blotting

Tissues were homogenized at 4°C in lysis buffer containing protease inhibitors. Then, the samples were centrifuged at 12,000×g for 15 min and the supernatants were collected. Proteins were separated by 10% SDS-polyacrylamide gel and transferred onto PVDF membranes. After blocking with 5% fat-free milk, the membrane was incubated with a primary antibody (TGF-β 1:300 dilution, CCN2 1:300 dilution, Santa Cruz, CA, USA) overnight at 4°C Then, the membrane were incubated with secondary antibodies conjugated to horseradish peroxidase for 1 h at 37°C. The proteins were visualized with an enhanced chemiluminescence reagent (Millipore, Billerica, MA, USA) after exposure to X-ray films. The densities of bands were analyzed by Quantity One and calculated by comparison to an internal control.

### RT-qPCR analysis

Total RNA from endometrial tissues was extracted using Trizol Reagent (Invitrogen) and reverse transcription reactions were performed using an RT Kit (Takara) according to the manufacturer’s instructions. cDNA was used as a template for PCR amplification of TGF-β and CCN2. Relative mRNA levels were evaluated by real-time quantitative RT-PCR with SYBR Green Master mix (Takra). GAPDH was used as an internal control. The primer sequences are shown in Table 3. The PCR conditions consisted of 5 min at 95°C for one cycle followed by 35 cycles of 95°C for 10 s, 60°C for 20 s, and 72°C for 20 s. The cycle threshold value was determined as the point at which the fluorescence exceeded a preset limit determined by the instrument’s software.

**Table 3:** The primer sequences.

Protein	Primer	Sequence	Fragment
TGF-β	Forward	5′-CTTCATGGTGGCTTTCTTCAA-3′	264
	Reverse	5′-CACTCCCCCTCACAGTAGTAG-3′	
CCN2	Forward	5′-GCTCCCTGCATCTTCGGTGGTAC-3′	298
	Reverse	5′-GGCAGTTGGCTCTAATCATAGTTGGG-3′	
GAPDH	Forward	5′-AACTTTGGTATCGTGGAAGGACTCA-3′	371
	Reverse	5′-GTGTCGCTGTTGAAGTCAGAGGAGA-3′	

### Immunohistochemistry

Tissues were fixed in neutral formaldehyde, embedded in paraffin and cut into 5-μm sections. Sections were subjected to routine immunohistochemical (IHC) staining as previously described[21]. Briefly, the sections were deparaffinized and rehydrated in a graded alcohol series, and antigen retrieval was performed in citrate buffer (pH 6.0) at 100°C for 15 min. After blocking with peroxide, the sections were incubated sequentially with a rabbit anti-human TGF-β polyclonal antibody (1:100, bs-0086R, Bioss, Beijing, China) and a goat anti-human CCN2 polyclonal antibody (1:100, sc-14939, Santa Cruz Biotechnology, Inc., USA) overnight at 4°C. Sections were incubated with the corresponding secondary antibodies conjugated to horseradish peroxidase at room temperature for 30 min. Finally, the sections were stained with diaminobenzidine (DAB), counterstained with hematoxylin, dehydrated, and cleared in xylene. As a negative control, the primary antibody was replaced with serum from non-immunized rabbit or goat.

### Cell culture

A human endometrial epithelial cell line (RL95-2) was purchased from the American Type Culture Collection (Rockville, MD, USA). RL95-2 cells were cultured in Dulbecco’s Modified Eagle’s Medium/F12 basal medium ((Sigma-Aldrich, St. Louis, MO, USA) with 10% fetal bovine serum ((Invitrogen, Carlsbad, CA, USA). The cell was incubated at 37°C in a 5% CO_2_ atmosphere.

### Statistical analysis

Statistical analyses were performed with the Statistical Package of Social Science 13.0 (SPSS, Inc., Chicago, IL, USA). One-way ANOVA followed by Tukey’ post hoc test and Student’s t-test were performed. Values are expressed as the mean ± SD. Correlation analysis was evaluated using the Pearson’s correlation test. A value of P < 0.05 was considered statistically significant.

## Results

### The expression of TGF-β and CCN2 in the endometrium of IUA patients

**Fig 1:**
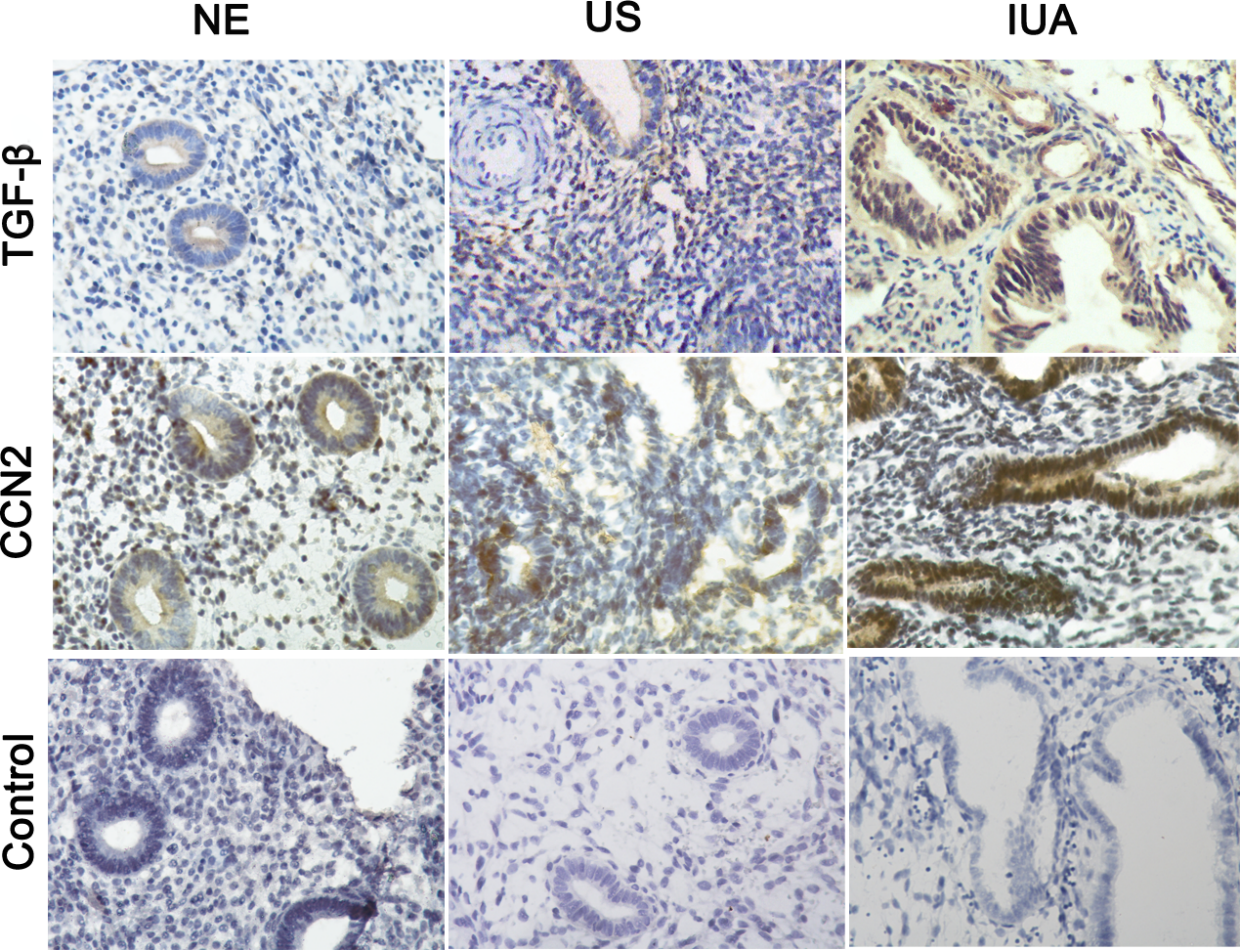
Immunohistochemical staining for TGF-β and CCN2 in endometrial tissue. 15 cases of normal endometrium, 15 cases of uterine septum and 70 cases of intrauterine adhesions were stained by immunohistochemistry, respectively. The representative localization of TGF-β and CCN2 expression is shown (magnification, 40×). The expression of TGF-β and CCN2 in IUA was higher than that seen in normal endometrium and uterine septum.

Immunohistochemical staining showed that TGF-β and CCN2 were primarily expressed in the cytoplasm and nucleus of epithelial and stromal cells located in the IUA endometrium. As shown in Fig 1, the expression of TGF-β and CCN2 in endometrial tissue of IUA was markedly increased compared to the normal endometrial tissue, suggesting that TGF-β and CCN2 maybe play an important role in IUA progression. Moreover, compared to the uterine septum, the expression of TGF-β and CCN2 in endometrial tissue of IUA was also increased.

**Fig 2:**
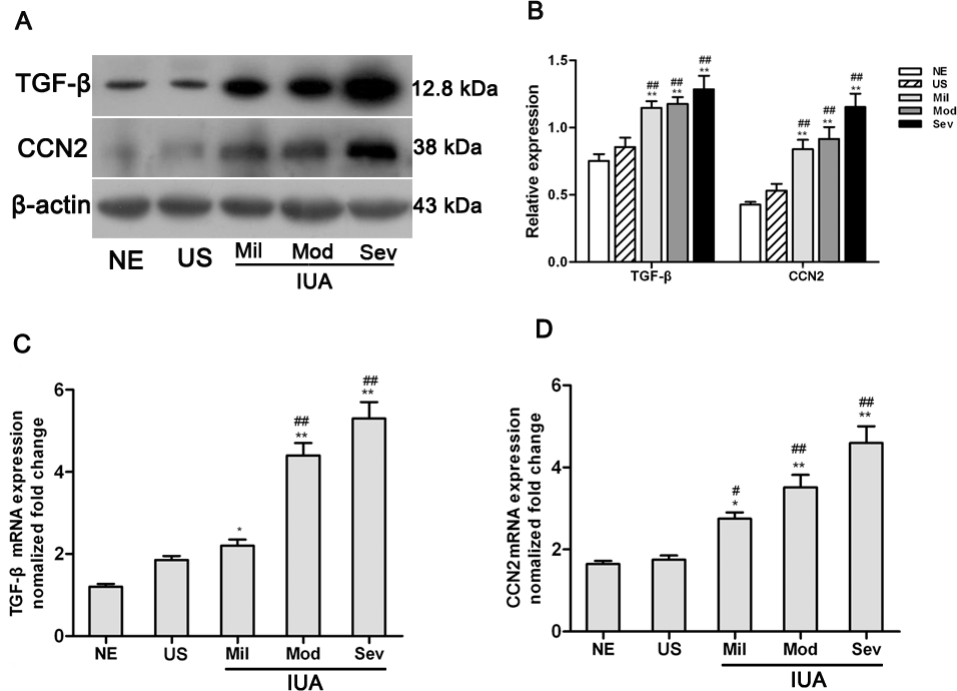
TGF-β and CCN2 were overexpressed in IUA endometrial tissue. (A) The expression of TGF-β and CCN2 in 15 cases of normal endometrium, 15 cases of uterine septum and 70 cases of intrauterine adhesions were measured by western blotting. Representative blots are shown. (B) A quantitative analysis of TGF-β and CCN2 expression in normal endometrium, uterine septum and intrauterine adhesions normalized to β-actin expression. The levels of TGF-β (C) and CCN2 (D) transcripts in 15 cases of normal endometrium, 15 cases of uterine septum and 70 cases of intrauterine adhesions were measured by qRT-PCR. Results are expressed as the mean±SD. One way ANOVA(Tukey’ post hoc test); * P<0.05, ** P<0.01 vs. normal endometrium, ^##^P<0.05, ^##^ P<0.01 vs. uterine septum.

To further confirm the expression of TGF-β and CCN2 in endometrium, a western blot assay was performed, with a representative blot shown in Fig 2A. The relative expression of both TGF-β and CCN2 proteins was calculated through normalization to β-actin, as summarized in Fig 2B. Similar to the results of immunohistochemical staining, TGF-β and CCN2 proteins were strongly expressed in IUA endometrium (Mild, Moderate and Severe groups) and weakly expressed in normal endometrium and uterine septum. A quantitative analysis of TGF-β and CCN2 expression revealed that TGF-β and CCN2 protein expression levels in IUA endometrial tissues were significantly increased compared to those in normal endometrial tissues and uterine septum (P<0.01).

In addition, consistent with the levels of protein expression, the levels of TGF-β and CCN2 transcript detected using qRT-PCR also showed that the expression of TGF-β and CCN2 mRNA in IUA endometrial tissues was significantly increased compared to that in normal endometrial tissues and uterine septum (P<0.01, Fig 2C and 2D). Most strikingly, the expression of TGF-β mRNA in the severe IUA group was approximately fourfold higher than in the normal endometrium group, and the expression of CCN2 mRNA was increased approximately threefold in the severe IUA group compared to the normal endometrium group. Collectively, these results demonstrate that TGF-β and CCN2 were highly expressed in IUA and may play an important role in IUA formation.

### NF-κB signaling pathway activity was enhanced in IUA endometrium

**Fig 3:**
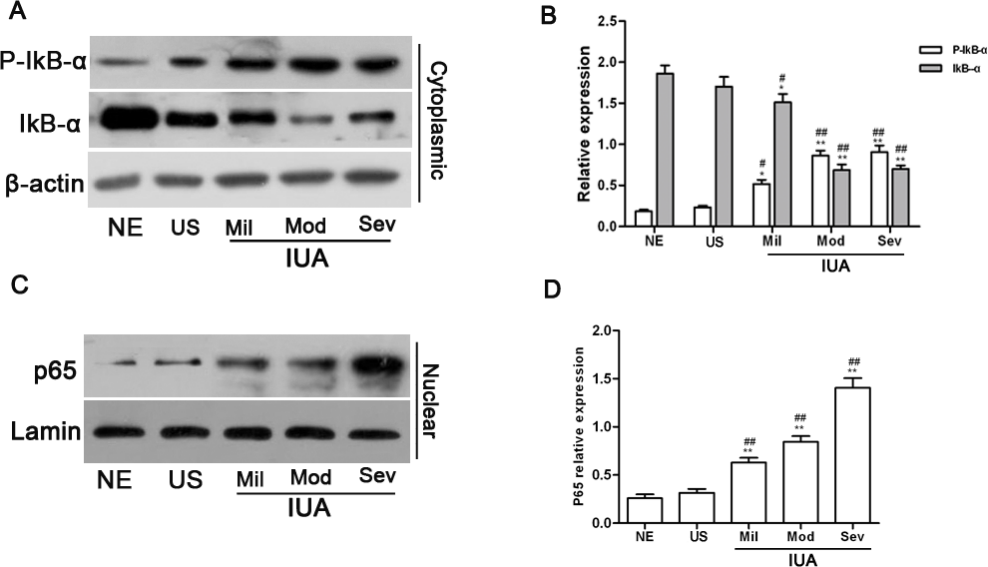
The activity of the NF-κB signaling pathway was enhanced in IUA endometrial tissue. IκB-α, p-IκB-α and p65 protein expression in 15 cases of normal endometrium, 15 cases of uterine septum and 70 cases of intrauterine adhesions were measured by western blotting. Representative blots are shown (A, C), and either β-actin or lamin was used as a loading control. (B, D) The relative expression of IκB-α, p-IκB-α and p65 in endometrial tissue was calculated through normalization to β-actin or lamin. Results are expressed as the mean±SD. One way ANOVA(Tukey’ post hoc test); * P<0.05, ** P<0.01 vs. normal endometrium, ^#^ P<0.05, ^##^ P<0.01 vs. uterine septum.

It has been reported that the NF-κB signaling pathway contributes to fibrogenesis[22]. However, the role of the NF-κB signaling pathway in IUA pathogenesis remains unclear. To clarify whether the NF-κB signaling pathway is involved in the formation of IUA, NF-κB signaling pathway activity in endometrial tissue was evaluated through the measurement of IκB-α, phosphorylated IκB-α(p-IκB-α) and p65 using western blotting. Representative blots are shown in Fig 3A and 3C. The relative expression of these proteins was calculated through normalization to β-actin or lamin, and are summarized in Fig 3B and 3D. The expression of IκB-α in the cytoplasm of IUA endometrial cells was significantly decreased compared to that seen in the normal endometrial tissue (P<0.01). In contrast, the expression of phosphorylated IκB-α(p-IκB-α) in the cytoplasm of IUA endometrial cells was significantly increased compared to normal endometrial tissue (P<0.01). Furthermore, the expression of p65 in the nuclei of IUA endometrial cells was markedly increased compared to that of the normal endometrial tissue (P<0.01). These data suggest that NF-κB signaling pathway activity in IUA endometrium was enhanced compared to normal endometrium.

Additionally, compared to the uterine septum, in which similar results were observed, the expression of IκB-α in the cytoplasm of IUA endometrial cells was significantly lower (P<0.01), and the expression of p-IκB-α in the cytoplasm of IUA endometrial cells was significantly higher (P<0.01). p65 expression in the nuclei of IUA endometrial cells was also markedly higher than that seen in the uterine septum (P<0.01). However, when the expression of IκB-α, p- IκB-α and p65 in the uterine septum was compared with that of normal endometrial tissue, no significant differences were found, which suggests that the NF-κB signaling pathway in uterine septum endometrium may not have been activated.

### The expression TGF-β and CCN2 were positively correlated with NF-κB pathway activity in IUA endometrium

**Fig 4:**
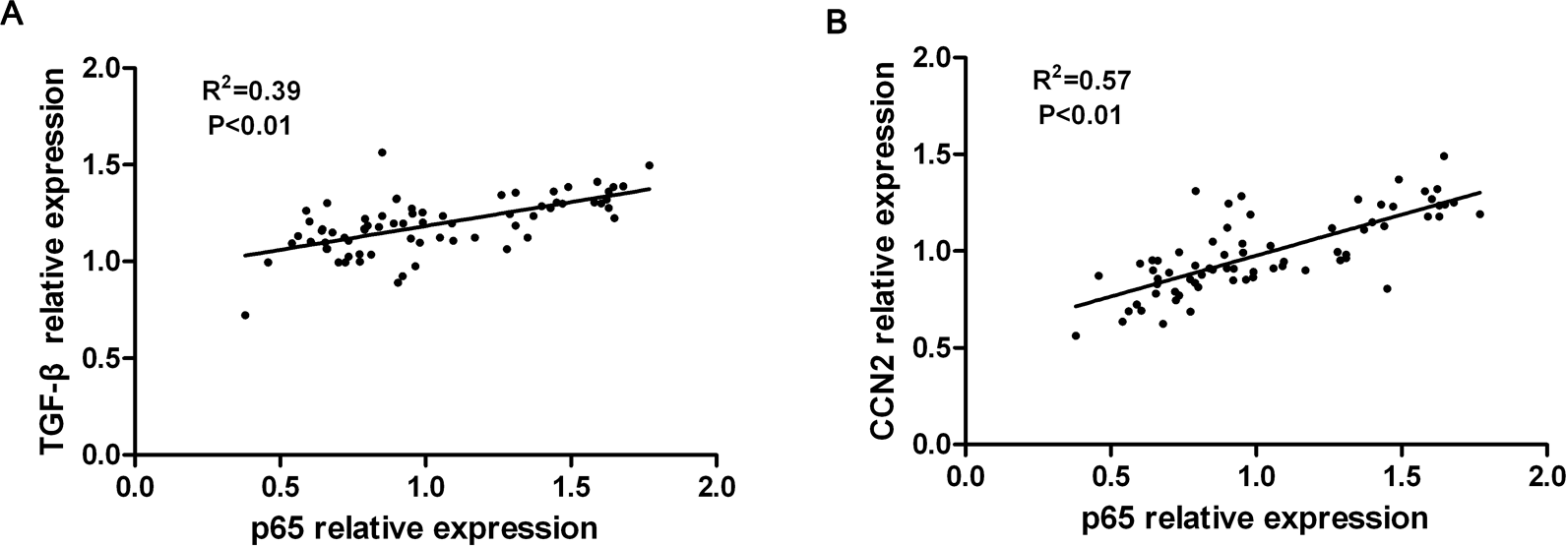
The expression TGF-β and CCN2 was positively correlated with p65 expression in IUA endometrial tissue. The correlation between TGF-β, CCN2 and p65 expression was analyzed using Pearson’s correlation test. (A) TGF-β expression was positively correlated with p65 expression in IUA endometrial tissue. (B) CCN2 expression was positively correlated with p65 expression in IUA endometrial tissue.

Recently, some reports have suggested that a crosstalk exists between TGF-β pathway and NF-κB pathways in pathological conditions [23]. In this study, we found that TGF-β and CCN2 expression and NF-κB pathway activity were all increased in IUA endometrial tissues. To test whether the expression of TGF-β and CCN2 was associated with the activation of the NF-κB signaling pathway in IUA endometrial tissues, the relationship between TGF-β/CCN2 and p65 expression was analyzed by Pearson’s correlation test. As shown in Fig 4, a positive correlation between TGF-β and p65 expression in the endometrial tissue of IUA was observed (R^2^ = 0.39, P<0.01). Additionally, CCN2 expression was also significantly correlated with p65 expression in IUA endometrial tissue (R^2^ = 0.57, P<0.01). These results suggest that the increased expression of TGF-β and CCN2 was likely associated with the activation of the NF-κB signaling pathway in IUA endometrium.

### The inhibition of the NF-κB signaling pathway attenuated the expression of TGF-β in RL95-2 cells

**Fig 5:**
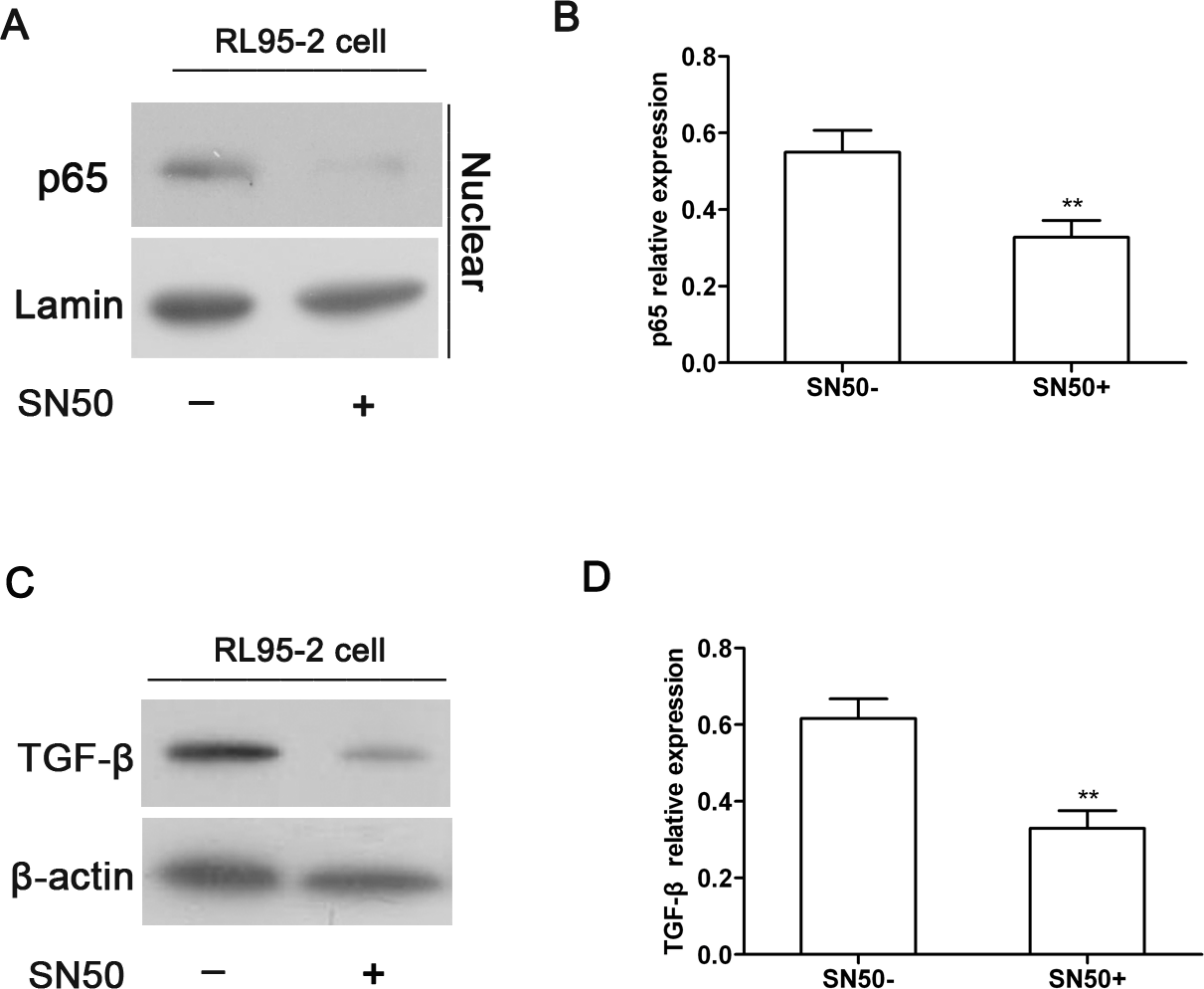
The inhibition of the NF-κB signaling pathway attenuated the expression of TGF-β in RL95-2 cells. RL95-2 cells were treated with SN50 (30 μg·ml^-1^), an inhibitor of the NF-κB signaling pathway, and the expression of p65 and TGF-β was measured by western blotting. (A) A representative blot and (B) quantitative analysis of p65 expression. (C) A representative blot and (D) quantitative analysis of TGF-β expression. Results were expressed as the mean±SD of three independent experiment performed in triplicate, ** P<0.01 vs.SN50(-).

To further confirm the correlation between the expression of TGF-β and the NF-κB signaling pathway in the endometrial tissues of IUA, we observed the expression of TGF-β in cultured RL95-2 cells after blocking the NF-κB signaling pathway using SN50, an inhibitor of the NF-κB signaling pathway. The expression of p65 and TGF-β proteins was measured by western blotting. Representative blots are shown in Fig 5A and 5C, and the relative expression of these proteins was calculated through gray-scale analysis.

As shown in Fig 5A and 5B, SN50 treatment resulted in a significant decrease in the level of nuclear p65 expression in RL95-2 cells, which suggests that the NF-κB signaling pathway was inhibited in RL95-2 cells. Accordingly, reduced expression of TGF-β in SN50-treated RL95-2 cells was also observed compared to the RL95-2 cells without SN50 treatment (P<0.01, Fig 5C and 5D), suggesting that blocking the NF-κB signaling pathway could attenuate the expression of TGF-β in endometrial cells. Therefore, these data further confirmed the above observation that TGF-β expression was positively associated with the activation of the NF-κB signaling pathway in IUA endometrial tissue.

### IUA recurrence was associated with levels of TGF-β and CCN2 expression

It is well established that adhesion recurrence after surgery is one of the most important factors underlying poor prognosis. To examine the risk factors for IUA recurrence, we investigated the recurrence rate in patients with IUA and post-uterine septum resection through follow-ups. As shown Table 4, the recurrence rates in patients with mild, moderate, and severe IUA were 23.07%, 44%, and 53.12%, respectively, whereas the recurrence rate in patients with post-uterine septum resection was 6.67%. The recurrence rate of IUA was significantly higher than that of post-uterine septum resection (P<0.01). The recurrence rate of IUA showed a dose-dependent relationship with the expression of TGF-β and CCN2. For example, IUA patients with high TGF-β and CCN2 expression were more likely to have a recurrence than IUA patients with lower TGF-β and CCN2 expression, suggesting that high TGF-β and CCN2 expression may be a potential predictor of the recurrence of IUA.

**Table 4:** The rate of recurrence in patients with intrauterine adhesions.

Group	n	Follow-up (years)	Recurrence (n)	Rate (%)
Uterine septum	15	2	1	6.67
Mild IUA	13	2	3	23.07
Moderate IUA	25	2	11	44.00
Severe IUA	32	2	17	53.12

## Discussion

IUA usually occurs after mechanical or infectious injury to the endometrium. For this reason, trauma and infection have been considered the most common causes of IUA. Any trauma that destroys the endometrial basal layer may result in IUA, including abortion, curettage, and hysteroscopic surgery, among others. However, the exact molecular mechanisms underlying the formation of IUA are still not fully understood. The current clinical treatment for IUA can restore the shape of the uterine cavity, but the repair of uterine physiological function remains difficult. Thus, it is necessary to investigate the molecular mechanisms of IUA genesis. In the present study, we first found that TGF-β and CCN2 were overexpressed in the endometrium of IUA, which was associated with the activation of NF-κB signaling pathway.

TGF-β and CCN2 have been shown to be involved in fibrogenesis[24, 25], and fibrosis plays a critical role in the pathogenesis of IUA. TGF-β plays an important role in modulating fibroblast phenotype and function, inducing myofibroblast transdifferentiation[26], and CCN2 has been shown to promote proliferation and extracellular matrix production in connective tissue[27].

The results of immunohistochemistry and western blot assays demonstrated that the protein expression of TGF-β and CCN2 in IUA endometrium was significantly increased compared to that seen in normal endometrium. Additional measurements of TGF-β and CCN2 transcripts also confirmed the high expression of TGF-β and CCN2 in the endometrium of IUA. Moreover, the level of TGF-β and CCN2 expression appeared to increase in parallel with IUA progression. These results suggested that the expression of TGF-β and CCN2 is associated with IUA, and may play an important role in IUA formation.

Previous studies have demonstrated that crosstalk between the TGF-β pathway and many other pathways, including the wnt/β-catenin, lysophosphatidic acid and NF-κB pathways, occurs during fibrogenesis[28–30]. In particular, evidence has accumulated showing that the NF-κB signaling pathway is involved in disorders of fibrogenesis[31]. In this study, the reduced expression of IκB-α and increased expression of p-IκB-α were found in IUA endometrial tissue compared to the normal endometrium. Furthermore, the level of p65 expression in IUA endometrial cell nuclei was also significantly increased compared to normal endometrium. These results suggest that the NF-κB signaling pathway was activated in IUA endometrium and also imply a critical role for the NF-κB signaling pathway in the formation of IUA.

Additionally, we found that the expression of TGF-β and CCN2 was positively correlated with p65 expression through correlation analysis, which suggests that the increased expression of TGF-β and CCN2 were likely associated with the activation of the NF-κB signaling pathway. To evaluate the effect of the NF-κB signaling pathway on TGF-β expression, the NF-κB signaling pathway was blocked using SN50, an inhibitor of the NF-κB signaling pathway, in RL95-2 cells. Western blotting showed that the inhibition of the NF-κB signaling pathway attenuated the expression of TGF-β in RL95-2 cells, suggesting that the expression of TGF-β in the endometrium of IUA could be induced by the NF-κB signaling pathway. Our findings are consistent with previous reports that the NF-κB signaling pathway was involved in TGF-β-induced β-catenin expression in human lung fibroblasts[31]. Moreover, other groups have reported that CCN2 could be induced by TGF-β and considered CCN2 to be a downstream mediator of the effects of TGF-β on fibroblasts[32]. Collectively, these data suggest that CCN2 and TGF-β expression in IUA endometrium are associated with the NF-κB signaling pathway.

Additionally, we found the rate of IUA recurrence in patients gradually increased from uterine septum through mild, moderate and severe IUA, which was consistent with the level of TGF-β and CCN2 expression in the endometrial tissues of uterine septum, mild, moderate and severe IUA. The recurrence rate of IUA showed a dose-dependent relationship with the expression of TGF-β and CCN2, suggesting that TGF-β and CCN2 are likely potential predictors of IUA recurrence.

In summary, we demonstrated for the first time that TGF-β and CCN2 are overexpressed in IUA endometrial tissue by a mechanism associated with the NF-κB signaling pathway. We hypothesize that the activation of the NF-κB signaling pathway may induce the expression of TGF-β and then cause the expression of CCN2, which ultimately results in the formation of IUA through the promotion of fibrotic processes. However, further studies are required to elucidate the exact underlying molecular mechanisms.

## Supporting Information

S1 Table Relative expression of TGF-β, CCN2, p-IκB-α, IκB-α and p65 in endometrial tissues measured by western blotting. (XLS)
